# Pneumonia amidst the COVID‐19 pandemic in Africa: Challenges and possible solutions

**DOI:** 10.1002/hsr2.493

**Published:** 2022-01-10

**Authors:** Olivier Uwishema, Helen Onyeaka, Baha Aldeen Abdalaziz Alshareif, Mohammed Eltahier Abdalla Omer, Alfredo Lorenzo Recio Sablay, Rabeet Tariq, Rayan Ibrahim Hamid Mohamed, Amirsaman Zahabioun, Mohamed Yousif Elamin Yousif, Elie Chalhoub, Marcos Roberto Tovani‐Palone

**Affiliations:** ^1^ Oli Health Magazine Organization, Research and Education Kigali Rwanda; ^2^ Clinton Global Initiative University New York New York USA; ^3^ Faculty of Medicine Karadeniz Technical University Trabzon Turkey; ^4^ School of Chemical Engineering University of Birmingham Edgbaston UK; ^5^ Faculty of Medicine and Surgery Al‐Zaiem Al‐Azhari University Khartoum Sudan; ^6^ Gadarif University Faculty of Medicine and Health Sciences Khartoum Sudan; ^7^ Faculty of Medicine and Surgery University of Santo Tomas Manila Philippines; ^8^ Liaquat National Hospital and Medical College Karachi Pakistan; ^9^ Faculty of Medicine University of Khartoum Khartoum Sudan; ^10^ College of Arts and Sciences: Department of Biology University of North Carolina at Chapel Hill Chapel Hill North Carolina USA; ^11^ Faculty of Medicine University of Saint Joseph of Beirut Beirut Lebanon; ^12^ Ribeirão Preto Medical School University of São Paulo Ribeirão Preto Brazil

Africa has been significantly affected by the ongoing coronavirus disease 2019 (COVID‐19) pandemic. It is noteworthy in this context that this is the continent with the poorest and most vulnerable populations to infectious diseases.^1^ Since the emergence of the pandemic, scientific journals have published much literature on the topic; however, among the research articles published from June to December 2020, only 1.5% mentioned Africa in their titles or abstracts. Moreover, data from Sub‐Saharan Africa underestimates the pandemic due to several factors, such as the overall low testing capacity and lack of laboratories, limited access to and weakness of health services, and political constraints, all of which suggest an imperative need for better reporting and further research on COVID‐19 in Africa.^2^

Pneumonia is the leading global cause of mortality in children under 5 years old. However, this disease still occurs frequently in working‐age immunocompromised individuals and older adults (>70 y/o) with prior chronic conditions.^3^, ^4^, ^5^ For decades, pneumonia has been the second‐leading cause of admission to adult medical wards in Africa, behind only malaria.^6^ Recently, although improved conjugate vaccines, such as the pneumococcal conjugate vaccine (PCV) and Haemophilus influenzae type b (HiB), have contributed to a decrease in the incidence and severity of pneumonia in infants and adults,^7^ the occurrence of secondary bacterial pneumonia, and fungal and viral pulmonary co‐infections on top of COVID‐19 pneumonia has worsened the situation.^8^, ^9^

Co‐infection with viral pneumonia is not rare^10^ and often results in hypoxia, acute respiratory distress syndrome, and multiple organ failure, with significant morbidity and mortality rates.^11^, ^12^ Despite this, no prevalence studies of pneumonia as a comorbidity of COVID‐19 or non‐COVID pneumonia have been conducted in Africa during the pandemic. Co‐infection with bacteria or fungi may complicate existing viral pneumonia, especially in critically ill patients.^13^ These infections may vary according to the local endemic/epidemic infections, suggesting that results may differ in Africa. Therefore, the continent's response to the pandemic should always consider the epidemiology of comorbidities and co‐infections.^14^ Currently, there is also a need for prevalence studies to define the significance of different types of pneumonia as a comorbidity of COVID‐19 or as individual infections apart from COVID‐19, followed by their respective morbidity and mortality status. Defining the significance of the problem would be only the first step in taking significant steps moving forward for its solution.

In addition to pneumonia, some African countries have faced other infectious diseases outbreaks.^9^ In light of this, new strategies to address risk factors for pneumonia and severe COVID‐19, such as child, maternal, environmental, pathogen, and health system factors, with a focus on strengthening opportunities for health promotion and infection prevention and control should primarily be explored.^4^ This can be initiated through supported community‐based healthcare strategies in health education and health promotion campaigns concerning hygiene, pneumonia, and COVID‐19. Moreover, assessing the knowledge, attitude, and perceptions of local households and communities towards pneumonia and COVID‐19, including other pertinent endemic infectious diseases, and especially tackling misinformation and vague practices can assist in developing specific and targeted measures for health promotion and education. This is because health measures must only be effective if the population would agree, cooperate, and be fully involved in their planning and implementation.^15^

Another critical point is that Pneumococcal conjugate vaccine (PCV) and Haemophilus influenzae type b (HiB) vaccination were shown to significantly decrease the incidence and severity of pneumonia‐related mortality in children.^4^, ^16^ With PCV vaccination specifically, it was estimated that pneumonia mortality was reduced by 23% to 33% in children less than 19 years old, preventing approximately 18 000 deaths between 2009 and 2016 in South Africa.^16^ Moreover, a significant decrease in pneumococcal pneumonia in adults has been achieved by the process of immunizing children, given that it interrupts the transmission of disease‐causing serotypes from the pediatric nasopharynx to susceptible adult populations.^4^ In this connection, it is beneficial to countries with high incidence and mortality of pneumonia to strengthen immunization programs for HiB, PCV, diphtheria, pertussis, measles, as well as Influenza, in line with the World Health Organization's Expanded Program on Immunization (EPI) programs, and thus to prevent pneumonia in relation to the capacity of each country.

Along with immunization, the inclusion of nutritional rehabilitation, zinc supplementation, exclusive breastfeeding, availability of drinking water and basic sanitation, and hygiene strategies will strengthen public health intervention for pneumonia prevention, especially amidst the COVID‐19 pandemic.^17^, ^18^, ^19^ In the settings that bear an additional burden of the pandemic, there must be availability and access to necessary antimicrobials and other medications in health care centers and hospitals, while simultaneously increasing the capacity and knowledge of community health workers and medical professionals.^7^ Intervention for respiratory care of severe COVID‐19 pneumonia patients as well as post‐intensive care programs must also be considered in this context.^20^

Africa is plagued by a weak health care system associated with a significant immunocompromised population.^1^ Harsh living conditions coupled with large families confined in limited living spaces should warrant a review of current containment, lockdown, and curfew implementations.^15^ To secure and sustain reduced morbidity and mortality of pneumonia, opportunities to improve the socioeconomic and living conditions of African communities must be explored.^4^ In addition, guidance and support from authorities and health agencies, transparent consultation and information dissemination regarding the COVID‐19 pandemic, as well as on other health concerns, would allow the population to make informed decisions to protect their health and those of their families.^15^

Finally, it is also worthwhile to promote community engagement through cultural and religious leaders and enable risk communication in local languages to rapidly identify red flag symptoms of COVID‐19 pneumonia, especially in local communities. Furthermore, telemedicine may be applied to reach patients in rural settings, where healthcare may be scarce, improving infectious disease surveillance.

## FUNDING

We did not receive any funding for this project.

## CONFLICT OF INTEREST

The authors declare no conflicts of interest.

## AUTHOR CONTRIBUTIONS

Conceptualization: Olivier Uwishema.

Data Curation: Olivier Uwishema, Helen Onyeaka, Baha Aldeen Abdalaziz Alshareif, Mohammed Eltahier Abdalla Omer, Alfredo Lorenzo Recio Sablay, Rabeet Tariq, Rayan Ibrahim Hamid Mohamed, Amirsaman Zahabioun, Mohamed Yousif Elamin Yousif, Elie Chalhoub, Marcos Roberto Tovani‐Palone.

Formal Analysis: Olivier Uwishema, Helen Onyeaka, Baha Aldeen Abdalaziz Alshareif, Mohammed Eltahier Abdalla Omer, Alfredo Lorenzo Recio Sablay, Rabeet Tariq, Rayan Ibrahim Hamid Mohamed, Amirsaman Zahabioun, Mohamed Yousif Elamin Yousif, Elie Chalhoub, Marcos Roberto Tovani‐Palone.

Methodology: Olivier Uwishema.

Project Administration: Olivier Uwishema.

Supervision: Marcos Roberto Tovani‐Palone.

Writing – Original Draft Preparation: Olivier Uwishema, Helen Onyeaka, Baha Aldeen Abdalaziz Alshareif, Mohammed Eltahier Abdalla Omer, Alfredo Lorenzo Recio Sablay, Rabeet Tariq, Rayan Ibrahim Hamid Mohamed, Amirsaman Zahabioun, Mohamed Yousif Elamin Yousif, Elie Chalhoub, Marcos Roberto Tovani‐Palone.

Writing – Review & Editing: Marcos Roberto Tovani‐Palone, Helen Onyeaka.

All authors have read and approved the final version of the manuscript.

## TRANSPARENCY STATEMENT

The authors affirm that this manuscript is an honest, accurate, and transparent account of the study being reported; that no important aspects of the study have been omitted; and that any discrepancies from the study as planned (and, if relevant, registered) have been explained.

## Data Availability

Data sharing is not applicable to this article as no new data were created or analyzed in this study.
